# Hypertension prevalence and influence of basal metabolic rate on blood pressure among adult students in Bangladesh

**DOI:** 10.1186/s12889-017-4617-9

**Published:** 2017-07-25

**Authors:** Nurshad Ali, Shakil Mahmood, M. Manirujjaman, Rasheda Perveen, Abdullah Al Nahid, Shamim Ahmed, Farida Adib Khanum, Mustafizur Rahman

**Affiliations:** 1 Department of Biochemistry and Molecular Biology, Shahjalal University of Science and Technology, Sylhet, 3114 Bangladesh; 2 Department of Biochemistry and Molecular Biology, Gonoshasthaya Samaj Vittik Medical College, Gono University, Savar, Dhaka, -1344 Bangladesh; 3 Department of Physiology, Gonoshasthaya Samaj Vittik Medical College, Gono University, Savar, Dhaka, -1344 Bangladesh

**Keywords:** Hypertension, Basal metabolic rate, Body mass index, Bangladesh

## Abstract

**Background:**

Hypertension is a global health issue and is currently increasing at rapid pace in South Asian countries including Bangladesh. Although, some studies on hypertension have been conducted in Bangladesh, there is a lack of scientific evidence in the adult student population that was missing from the previous and recent national cross-sectional studies. Moreover, the specific risk factors of hypertension in the Bangladeshi adults still need to be investigated. This study was conducted to estimate hypertension prevalence among adult students in Bangladesh and to test the hypothesis of Luke et al. (Hypertension 43:555–560, 2004) that basal metabolic rate (BMR) and blood pressure are positively associated independent of body size.

**Method:**

The data was collected on 184 adult university students (118 female and 66 male) in Dhaka, Bangladesh. Anthropometric, BMR details and an average of at least two blood pressure measurements were obtained. Hypertension was defined by a systolic blood pressure (SBP) ≥ 140 mmHg and/or, diastolic blood pressure (DBP) ≥ 90 mmHg.

**Results:**

Overall, 6.5% of participants had hypertension with significantly (*p* < 0.001) higher prevalence in male (12.1%) than in the female (3.4%) students. Age and BMI showed positive and significant correlation with hypertension among the students. When adjusted for body mass index (BMI), as well as other potentially confounding variables such as age, sex, smoking status and degree of urbanization, BMR was positively correlated with SBP and DBP (*p* < 0.001). Thus, higher BMR is associated with SBP and DBP; this is opposite the well documented inverse relationship between physical activity and blood pressure. If the influence of BMR on blood pressure is confirmed, the systematically elevated BMR might be an important predictor that can explain relatively high blood pressure and hypertension in humans.

**Conclusion:**

This study reports the prevalence and associated risk factors of hypertension in the Bangladeshi adult students. The study also showed a positive association between BMR and blood pressure among the participants. A large scale longitudinal study across the country is needed to find out the underlying causes of hypertension in the Bangladeshi adults. In addition, comprehensive and integrated intervention programs focusing on modifiable risk factors are recommended to make awareness and prevent hypertension.

## Background

Hypertension is a global health issue and has been recognized as a major contributor to the burden of cardiovascular diseases (CVDs), kidney failure and premature death [3, 15, 47]. According to the World Health Organization (WHO), about 17 million deaths occur worldwide due to CVDs of which hypertension and its complications account for an estimated 9.4 million deaths [48, 28] and a major portion of CVDs related deaths (80%) occurred in the developing countries [30]. The prevalence of hypertension including CVDs are increasing in Asia especially in Southeast Asian countries [38, 44, 17, 40]. In Asian region, hypertension is counting as a significant health concern, affecting more than one third of the adult population [35]. The two fast-growing economies China and India are facing the burden of hypertension and are projected to proliferate by 2025 [23]. Bangladesh is a developing country in Southeast Asia, having experience with the epidemiological transition of communicable diseases to non-communicable diseases [19]. Epidemiological studies in South Asia have shown that life style changes associated with rapid urbanization, less physical activity, low intake of vegetables and fruits have led to increase the rate of CVDs including hypertension in Bangladesh [20]. Previously, a meta-analysis covering studies up to 1994 indicated the prevalence of hypertension 11.3% in the adult population of Bangladesh [51]. Later on, few small scale studies also reported the prevalence of hypertension ranging from 11 to 44% in the Bangladeshi population [22, 32, 39]. A more systematic review and meta-analysis of the prevalence of hypertension among 6430 Bangladeshi adults for the period 1995 to 2009 was estimated to be 13.5% ranging from 12.7 to 14.3% [33]. A recent national cross-sectional study among the Bangladeshi adults aged 35 years to older documented the prevalence of hypertension 26.4% with higher incidence 32.4% in women than 20.3% in men [7]. However, adult student population (age range 18–25 years) was not included in the recent national cross-sectional study and other previous studies in Bangladesh.

It is well known that, the consequences of high blood pressure include heart failure, coronary heart disease, peripheral arterial disease, stroke, and kidney disease [6, 21]. A number of risk factors are associated with the development of hypertension, including less physical activity, excess body fat, intake of high dietary sodium and low potassium, more alcohol consumption, and chronic psychosocial stress; these risk factors, however, cannot fully explain the variation in blood pressure and hypertension prevalence that occurs within and between human populations [1, 13, 18, 42]. At present, the specific causes of hypertension are unknown in nearly 90–95% individuals [13, 21]. Therefore, more research is required to find out actual causes of hypertension in human populations.

Studies among Nigerians and African Americans have suggested a link between maintenance energy costs and blood pressure; after adjusting for the effects of age, body size, and body composition, BMR exerts a positive influence on both systolic blood pressure (SBP) and diastolic blood pressure (DBP) [31]. Another study among indigenous Siberians has shown that after adjusting body size and composition, as well as potentially confounding variables such as age, smoking status, ethnicity, and degree of urbanization, BMR is positively correlated with SBP and pulse pressure (PP) and a positive trend with DBP [42]. A recent study showed a positive and significant association between basal metabolic rate and blood pressure in Asian women [41]. This link, if confirmed, may shed light on global variation in blood pressure, given the presence of interpopulation differences in BMR [8, 26, 27, 42].

In Bangladesh, data on hypertension prevalence in the adult student population are missing from previous studies and the associated factors of hypertension still need to be investigated. Therefore, we conducted a cross-sectional study with specific aims. First, to estimate the prevalence of hypertension and find out the possible risk factors of hypertension among adult students (aged 18–25 years) in Bangladesh and secondly, to test the hypothesis of Luke et al. [31] that BMR and blood pressure are positively correlated independent of age, body size and composition, and selected lifestyle variables.

## Methods

### Study population

This study was carried out at the Gonoshasthaya Samaj Vittik Medical College, located of Savar region in the Dhaka district. The study population consisted of male and female students (aged between 21 and 23 years) who were studied at the first year of honours of MBBS (Bachelor of Medicine and Bachelor of Surgery).

### Sampling design and sample size

This study was cross-sectional design and data was collected between June and August 2012. A total of 184 healthy adult students (122 females and 66 males) were selected from two semesters at first year of honours level. All participants gave their written consent before inclusion in the study which was approved by the Ethics Committee of the Medical College. Anthropometric data, blood pressure and BMR measurements were recorded by trained health technicians at Biochemistry and Physiology department of the Medical College. The quality of anthropometric data ensured by repeated measurements in presence of investigators. All participants provided their individual anthropometric and life style information in a structured questionnaire form.

### Anthropometry and basal metabolic rate (BMR)

Anthropometric data such as height, weight, three circumferences and skinfold thickness at four sites (biceps, triceps, subscapular and suprailaic) were obtained using the standard procedure [29]. Individual body composition was assessed using two derived measures: BMI (Body mass index; kg/m^2^) and BF (body fat) percentage. Percentages of body fat, basal metabolic rate (BMR) and body mass index (BMI) were measured using an Omron body fat analyzer (Omron Corporation, Tokyo, Japan). Fat-free mass (FFM) was calculated as body mass less fat mass. All participants rested quietly in a supine position for a minimum of 10 min before BMR measurement. Following [42] study, all BMR measurements were done under standard conditions, i.e. in a quite environment and at a room temperature of about 25 °C with the study subjects in a postabsorptive condition after a 12 h fast. All participants were familiarized with the equipment and procedure before BMR measurements to reduce anxiety. BMR was measured by inserting of individual age, sex and anthropometric data into the equipment. The apparatus was routinely checked by burning ethanol and BMR measurements were validated by inter-day assays. Measured BMR was compared with predicted values based on standards for body mass and FFM. Based on the sex- and age-specific Oxford predictive equations of Henry [16], BMR standards for body mass were measured and predicted BMR was from FFM based on the general predictive equation of Cunningham [9].

### Blood pressure variables

The participants were allowed 10 min rest before measuring blood pressure two times at 5 min intervals on the left arm in a sitting position with the arm supported at the level of the heart using an Omron M10 digital BP machine (Omron Corporation, Tokyo, Japan). The mean value of the first and second measurements was considered for systolic blood pressure (SBP) and diastolic blood pressure (DBP). Blood pressure measurements taken from all participants in the morning 8:30 to 10:30 am. Pulse pressure (PP), which serves as an indicator of arterial stiffness and blood vessel wall inflammation and is a predictor of cardiovascular disease mortality [14, 21], was calculated as the difference between SBP and DBP. SBP ≥ 140 mmHg or DBP ≥ 90 mmHg or both were defined as hypertension.

### Statistical analysis

All data were analyzed using the software IBM SPSS statistics version 23. Independent sample *t*-test (two tailed) was done to assess the differences between males and females for anthropometric, blood pressure and metabolic variables. Measured versus predicted BMR (for body mass and FFM) was compared using paired-sample *t*-tests (two tailed). Interrelationships between anthropometric, blood pressure and metabolic variables were assessed by Pearson’s correlation coefficient test. Linear regression analyses with blood pressure measures as the dependent variable (SBP, DBP, and PP) were used to estimate the relative contribution of BMR to blood pressure while considering sex, age, BMI, degree of urbanization (i.e., residence in village vs. town) and smoking status (i.e., active smoker vs. nonsmoker). The values in tables were presented as mean ± standard deviation otherwise noted. A level of alpha 0.05 was assigned for statistical significance.

## Results

### Baseline characteristics and blood pressure data

**Fig. 1:**
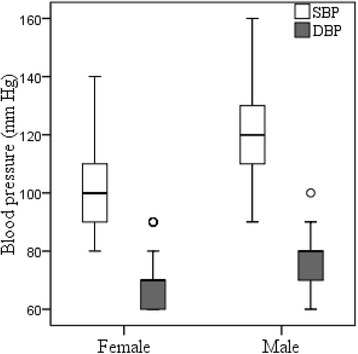
Level of systolic and diastolic blood pressure (mm Hg) in male-female group

Anthropometric and blood pressure and metabolic data for the total sample (118 females and 66 males) are presented in Table 1. The mean age of the female students was 19.8 ± 0.6 years (range: 18–21 years), and 20.4 ± 1.0 years (range: 18–223 years) for males. The average BMI for all subjects was 22.3 ± 3.7 kg/m^2^. Males (22.8 ± 3.6 kg/m^2^) had slightly higher BMI than females (22.1 ± 3.7 kg/m^2^). According to WHO [50] categories, 4.5% of individuals were classified as obese (i.e., BMI ≥ 30), 18.2% as overweight (BMI 25–29.9), 63.7% as normal (BMI < 25) and 13.6% as underweight (BMI <18.5) among males students (Table 2). Among female students, 3.4% of individuals were classified as obese, 14.4% as overweight, 66.8% as normal and 15.5% as underweight (Table 2). The average level of SBP and DBP was significantly (*p* < 0.001) higher in male students (121 ± 13 mmHg; 76 ± 8 mmHg, respectively) than in the female students (105 ± 13 mmHg: 68 ± 7 mmHg, respectively) (Table 1 and Fig. 1). According to categories by Chobanian et al. [6] and WHO/FAO [49], among male students, 12.1% of individuals were classified as hypertensive (SBP ≥ 140 or DBP ≥ 90 mmHg), 56.1% as prehypertensive (SBP 120–139 or DBP 80–89 mmHg), and 32.8% as normal (SBP < 120 and DBP < 80) (Table 2). Among the female students, 3.4% of individuals were classified as hypertensive, 18.6% as prehypertensive and 78% as normal (Table 2).

**Table 1:** Descriptive characteristics for anthropometric and blood pressure data

Measure	Total (*n* = 184)	Female (*n* = 118)	Male (*n* = 66)
Age (years)	20.0 ± 0.8 (18–23)	19.8 ± 0.6 (18–21)	20.4 ± 1.0 (18–23)^**^
Height (cm)	159.1 ± 8.8	154.2 ± 5.4	168.0 ± 6.6^**^
Body mass (kg)	56.8 ± 11.7	52.5 ± 9.5	64.5 ± 11.2^**^
Sum of skinfolds (mm)	63.9 ± 24.6	82.3 ± 25.6	45.6 ± 22.4^**^
Body fat (%)	19.8 ± 0.6	28.1 ± 6.3	20.2 ± 5.5^**^
Fat-free mass (kg)	42.2 ± 8.4	37.3 ± 4.1	51.1 ± 6.8^**^
Fat mass (kg)	14.6 ± 5.9	15.2 ± 6.0	13.5 ± 5.6^*^
BMI (kg/m^2^)	22.3 ± 3.7	22.1 ± 3.7	22.8 ± 3.6
SBP (mm Hg)	110 ± 15 (80–160)	105 ± 13 (80–140)	121 ± 13 (90–160)^**^
DBP (mm Hg)	71 ± 8 (60–100)	68 ± 7 (60–90)	76 ± 8 (60–100)^**^
PP (mm Hg)	39 ± 9 (20–60)	36 ± 8 (20–60)	44 ± 8 (30–60)^**^
Smoking status			
Yes	15	0	15^**^
No	169	118	51
Place of residence			
Urban	125	80	45
Rural	59	38	21

Values are presented as mean ± SD. Ranges for some variables are presented within first bracket. ^**^ *P* < 0.001 and ^*^ *P* < 0.05 when compared the mean value with female. *P*-values are obtained from independent samples *t*-test

**Table 2:** Categories of participants by body mass index (BMI) and hypertension prevalence

Measure	Total n (%)	Female n (%)	Male n (%)
Body mass index category			
Underweight	27 (14.7)	18 (15.5)	9 (13.6)
Normal	121 (65.7)	79 (66.8)	42 (63.7)
Overweight	29 (15.8)	17 (14.4)	12 (18.2)
Obese	7 (3.8)	4 (3.4)	3 (4.5)
Blood pressure category			
Normal	113 (61.4)	92 (78.0)	21(32.8)^*^
Prehypertensive	59 (32.1)	22 (18.6)	37 (56.1)^*^
Hypertensive	12 (6.5)	4 (3.4)	8 (12.1)^*^

BMI (kg/m^2^) was categorized as underweight (BMI <18.5), normal (BMI <25), overweight (BMI 25–29.9) and obese (BMI ≥ 30) according to WHO (2000). Blood pressure (mm Hg) was categorized as normal (SBP < 120; DBP < 80), Prehypertensive (SBP 120–139; DBP 80–89) and Hypertensive (SBP ≥ 140; DBP ≥ 90) according to Chobanian et al. [6]. ^*^ *P* < 0.001 when compared with female. *P*-values are obtained from independent samples *t*-test

### Interrelationships between metabolic, anthropometric and blood pressure variables

Measured and predicted BMRs (± SD) relative to body mass and FFM standards were recorded for all students (Table 3). The male students had significantly higher mean level of measured BMRs than female students (6457 ± 882 vs. 5163 ± 737 kJ/day, *p* < 0,001). Both male and female students did not show significant elevations in BMR over that predicted by body mass (6457 ± 882 vs. 6930 ± 715 kJ/day in males and 5163 ± 737 vs. 5650 ± 398 kJ/day in females). However, both male and female students showed significant elevations in BMR over predicted for FFM (6457 ± 882 vs. 6151 ± 487 kJ/day in males [*p* < 0.001] and 5163 ± 737 vs. 5039 ± 192 kJ/day in females [*p* < 0.05]).

**Table 3:** Measured BMR (mean ± SD) versus predicted BMR^a^

	Female (*n* = 118)	Male (*n* = 66)
BMR vs. body mass		
Measured (kJ/day)	5163 ± 737	6457 ± 882^**^
Predicted (kJ/day)	5650 ± 398	6930 ± 715
BMR vs. fat free mass		
Measured (kJ/day)	5163 ± 737^*^	6457 ± 882^**^
Predicted (kJ/day)	5039 ± 192	6151 ± 487

^a^Differences between males and females are significant at ^**^ *p* < 0.001. Differences between measured and predicted by fat free mass are significant at: ^*^ *p* < 0.05; ^**^ *p* < 0.001. *P*-values are obtained from paired-sample *t*-tests

Tables 4 and 5 summarizes the result from Pearson’s correlation coefficient test (two-tailed) used to assess the interrelationships between the anthropometric, metabolic and blood pressure variables in the total sample. When sexes is combined, age was positively correlated with SBP (*p* < 0.01), DBP (*p* < 0.05) and PP (*p* < 0.01). However, after independent sex analysis, age was not significantly correlated with SBP, DBP and PP in male-female subgroup (Tables 4 and 5). The influence of BMR on blood pressure was assessed initially with pair-wise correlation and subsequent analyses with linear regressions to adjust for potentially confounding factors. The initial analysis with combined sexes, BMR was positively correlated (*p* < 0.01) with SBP, DBP and PP. In subsequent analyses, BMR was significantly correlated with SBP (*p* < 0.001), DBP (*p* < 0.001) and PP (*p* < 0.01) in females (Table 4). Among males, BMR was significantly correlated with SBP (*p* < 0.01), DBP (*p* < 0.01) and PP (*p* < 0.05) (Table 5). Linear regression analysis was done to estimate the relative contribution of BMR to blood pressure variation. A regression model with age, sex, BMI, smoking status, urbanization and BMR showed 41% and 38% variations in SBP and DBP, respectively; BMR was a significant predictor (*p* < 0.001) of SBP and DBP in this model (Table 6).

**Table 4:** Correlation matrix for anthropometric, metabolic and blood pressure data for female students^a^

	BMR (KJ/day)	Age (years)	Body mass (kg)	BMI (kg/m^2^)	FFM (kg)	Fat mass (kg)	SBP (mm Hg)	DBP (mm Hg)	PP (mm Hg)
BMR (KJ/day)	1	0.097	0.998^**^	0.904^**^	0.931^**^	0.953^**^	0.466^**^	0.496^**^	0.300^**^
Age (years)		1	0.089	0.062	0.115	0.062	0.075	0.044	0.081
Body mass (kg)			1	0.912^**^	0.921^**^	0.963^**^	0.467^**^	0.491^**^	0.306^**^
BMI (kg/m^2^)				1	0.723^**^	0.958^**^	0.370^**^	0.407^**^	0.226^*^
FFM (kg)					1	0.783^**^	0.509^**^	0.516^**^	0.351^**^
Fat mass (kg)						1	0.395^**^	0.429^**^	0.248^**^
SBP (mm Hg)							1	0.822^**^	0.861^**^
DBP (mm Hg)								1	0.418^**^
PP (mm Hg)									1

^a^Correlations are statistically significant at: ^**^ *p* < 0.01; ^*^ *p* < 0.05

**Table 5:** Correlation matrix for anthropometric, metabolic and blood pressure data for male students^a^

	BMR (KJ/day)	Age (years)	Body mass (kg)	BMI (kg/m^2^)	FFM (kg)	Fat mass (kg)	SBP (mm Hg)	DBP (mm Hg)	PP (mm Hg)
BMR (KJ/day)	1	0.101	0.997^**^	0.885^**^	0.922^**^	0.865^**^	0.410^**^	0.406^**^	0.271^*^
Age (years)		1	0.086	0.114	0.100	0.050	0.049	0.033	0.046
Body mass (kg)			1	0.891^**^	0.918^**^	0.876^**^	0.401^**^	0.403^**^	0.259^*^
BMI (kg/m^2^)				1	0.692^**^	0.933^**^	0.347^**^	0.336^**^	0.235
FFM (kg)					1	0.612^**^	0.403^**^	0.370^**^	0.294^*^
Fat mass (kg)						1	0.310^*^	0.353^**^	0.158
SBP (mm Hg)							1	0.819^**^	0.830^**^
DBP (mm Hg)								1	0.357^**^
PP (mm Hg)									1

^a^Correlations are statistically significant at: ^**^ *p* < 0.01; ^*^ *p* < 0.05

**Table 6:** Linear regression model for prediction of blood pressure among the students (sexes combined)

Parameters	Coefficient (B)	*R*-square	*t*-value	*p*-value
Systolic blood pressure (SBP)		0.410		
Constant	52.832		2.339	<0.05
Sex^a^	−0.178		−0.087	0.930
Age (years)	0.663		0.580	0.563
BMI	−0.968		−2.571	0.011
Smoking status^b^	−4.978		−1.415	0.159
Urbanization^c^	−1.037		−0.547	0.585
BMR (KJ/day)	0.012		8.424	<0.001
Diastolic blood pressure (DBP)		0.380		
Constant	44.386		3.460	<0.01
Sex^a^	−0.649		−0.559	0.577
Age (years)	0.112		0.172	0.863
BMI	−0.369		−1.727	0.086
Smoking status^b^	−1.559		−0.780	0.436
Urbanization^c^	−1.135		−1.054	0.293
BMR (KJ/day)	0.006		7.528	<0.001

^a^Sex (0 = female; 1 = male)

^b^Smoking status (0 = no; 1 = yes)

^c^Urbanization (0 = village; 1 = town)

## Discussion

This is the first study that reports the hypertension prevalence and influence of BMR on blood pressure among adult students in Bangladesh. Among total sample, 6.5% of individuals were classified as hypertensive and this prevalence was higher in male (12.1%) than in the female (3.4%) participants (Table 2). The higher prevalence of hypertension has also been reported in adult male than in the adult female residents in rural areas in Bangladesh [24]. Another studies in Bangladesh, reported the high prevalence of hypertension in adult females than in the adult males [7, 33]. This variation of hypertension prevalence among male-female groups may depend on of age, food habits, physical exercise, dietary salt intake and smoking status. In present study, independent sex analysis showed that smoking is positively correlated with SBP and DBP among the male students (data not shown). We could not conduct such analysis for females, as in Bangladesh females are generally not used to smoking. Blood pressure level and rates of hypertension prevalence documented here are slightly lower (for females), or consistent (for males) with the finding observed in previous studies in Bangladesh [7, 33, 22, 32, 39].

Statistical analyses showed that age, higher BMI and body composition were significant factors associated with hypertension among the adult students (Tables 4 and 5), which are in line with the findings of previous studies in Bangladesh [7, 33]. A study by Chowdhury et al. [7] showed that age is one of important contributing risk factor of hypertension and the trends are more frequent at older stage among the Bangladeshi adults. We found a significant positive association between increasing age and hypertension considering all sample (data not shown). However, independent sex analysis did not show significant association between age and hypertension in male-female group. This finding could be a reason that all participants were adult students aged between 18 and 23 years and age differences within male-female group were not so pronounced. Age is unchangeable risk factor of hypertension [25]. In Bangladesh, due to the decline in fertility and a steady increase in life expectancy the population age structure is changing and the number of old age population will increase rapidly which will strengthen the hypertension risk among the older population in near future [7]. Therefore, other modifiable factors should be taken into consideration through intervention programs among the people. For example, increasing physical activity and cutting fatty food from daily menu could be an effective option [7].

In Bangladesh, industrialization and urbanization are increasing day by day. Recent studies showed that hypertension is more prevalent in urban people than in the rural people [2, 7, 24]. In this study, we did not find the significant positive association between degree of urbanization and hypertension. This could be a reason that major portion of the students (68%) included here are already from urban areas.

The present study results are consistent with previous finding of Snodgrass et al. [42] and Luke et al. [31] showing that BMR is positively associated with SBP and DBP measures independent of sex, age, body size and body composition among a large sample of indigenous Siberians (*n* = 284) and Nigerians (*n* = 996) and African Americans (*n* = 452), respectively. When adjusted body size and composition, as well as other potentially confounding variables such as age, degree of urbanization and smoking status, BMR was positively correlated with SBP and DBP (Table 6). Thus, this confirms the association of BMR with higher blood pressure. In fact, multiple regression analysis for both SBP and DBP showed that BMR is the strongest predictor of blood pressure. Therefore, we agree with Snodgrass et al. [42] finding that observed result suggest a casual relationship between BMR and blood pressure, however this cross sectional study does not allow conclusion to be drawn the causality.

If the influence of BMR on blood pressure is confirmed, it would represent an important step toward understanding the cardiovascular risk among the Bangladeshi adults. Even, relatively small differences in blood pressure level can have significant health consequences [42]. For example, a clinical study by Baker and Godfrey [5] suggest that lowering SBP by 10 mmHg can reduce total mortality risk by 30%. Moreover, the link between BMR and blood pressure could also explain a pathway linking biological adaptation to regional environment conditions with changes of health status associated with economic development [42]. This observation is important to understand the role of underlying biological differences between populations and in structuring variations in chronic disease patterns reported in modernizing populations [11, 37, 45].

The mechanism responsible for the association between BMR and blood pressure is unknown at present. We agree with Snodgrass et al. [42] that few possible explanatory mechanisms could be considered here. First, according to Luke et al. [31], heightened sympathetic tone (ie, the background activity of the sympathetic nervous system [SNS] is associated with upregulated BMR which could elevate blood pressure. Moreover, the SNS including tonic sympathetic activity, is closely involved with energy regulation, and sympathetic stimulation can lead to a twofold increase BMR [13, 34]. According to Monroe et al. [34] variation in sympathetic b-adrenergic sensitivity and sympathetic tone may contribute to population differences in energy expenditure. Second, thyroid hormones (T_3_ and T_4_) may mediate the association between BMR and blood pressure. Clinical studies say that both hypothyroidism and hyperthyroidism can lead to elevated blood pressure, and there is plausible evidence that subclinical thyroid “dysfunction” accelerates the changes in cardiovascular function and blood pressure regulation [10, 12]. Moreover, thyroid hormone levels are closely related to BMR through their direct effects on rates of oxidative metabolism in most tissues [13]. Third, a potential mechanism linking BMR and blood pressure is oxidative stress, with increased formation of reactive oxygen species (ROS) heightening oxidative damage and precipating increases in blood pressure [42]. This evidence supports the role of ROS in the pathophysiology of hypertension through multiple mechanisms, including inflammatory processes, endothelial remodeling, renal dysfunction and altered nitric oxide regulation [36, 43]. Finally, developmental effects could have a link between adult blood pressure and BMR [42]. Studies documented the association of low birth weights with higher blood pressure later in life [1, 5] and these negative effects in health appear to be magnified when low birth weight is associated with rapid postnatal growth [1, 4]. Moreover, low birth weight is also associated with a relatively high resting heart rate and high sleeping metabolic rate [46], raising the intriguing possibility of developmental programming of metabolic regulation [42].

We hope that, this study finding will stimulate further research exploring the influence of BMR on blood pressure among the Bangladeshi adults. We agree with the author’s suggestions that awareness and simple health messages, increase provider visit, can reduce blood pressure and improve blood pressure control in hypertensive adults [2, 7].

### Strengths and limitations

The major strengths of our study were to estimate the hypertension prevalence in the adult student population (aged 18–23 years) that was missing from the national cross-sectional study in Bangladesh. The possible risk factors of hypertension have been discussed in the study. This study has also evaluated the association between BMR and blood pressure among the participants. Moreover, hypertension measurements were collected by trained and experienced health technicians using standard methods rather self-reporting. Therefore, the measurements error and bias is less in this study compared to other cross sectional studies in Bangladesh. The main limitation of our study was the small sample size (*n* = 118) and unequal male-female participants (88 males and 118 females) that do not represent all Bangladeshi adult students. In this study, we did not record information on the anti-hypertensive medication. Other limitations included missing information of some important factors of hypertension like physical exercise, family history of hypertension, salt intake and lipid profile of the participants.

## Conclusion

The present study reports a number of risk factors that is associated with hypertension among the Bangladeshi adult students. Statistical analysis also indicates a positive association between BMR and blood pressure among the participants. The number of young adults in Bangladesh is a significant proportion of total population in this country. Thus, hypertension prevalence in this special group is alarming. Since most of the factors associated with hypertension are preventable and modifiable. Therefore, the early diagnosis, life style changes, healthy food habits and overall awareness can reduce the rates of having hypertension in Bangladeshi population. Furthermore, a large scale longitudinal study across the country is required to find out the underlying causes of hypertension among Bangladeshi adults.

## Abbreviations

BMI: Body mass index
BMR: Basal metabolic rate
BP: Blood pressure
CVD: Cardiovascular disease
DBP: Diastolic blood pressure
PP: Pulse pressure
SBP: Systolic blood pressure
SD: Standard deviation
